# Disseminated Peritoneal Tuberculosis Initially Misdiagnosed as Nephrogenic Ascites

**DOI:** 10.1155/2023/4240423

**Published:** 2023-04-20

**Authors:** Lauren Crossman, Christopher Ronald Funk, Sheetal Kandiah, Reena Hemrajani

**Affiliations:** ^1^Emory University School of Medicine, Atlanta, GA, USA; ^2^Division of Infectious Diseases, Emory University School of Medicine, Atlanta, GA, USA; ^3^Division of Hospital Medicine, Emory University School of Medicine, Atlanta, GA, USA

## Abstract

A middle-aged immigrant male from a region with endemic tuberculosis who had a history of end-stage kidney disease presented to the emergency room for routine hemodialysis and abdominal swelling. He was admitted to the medicine service for suggested daily dialysis to improve his volume overload, which was attributed to nephrogenic ascites. He was found to have several findings concerning for systemic illness, including fevers, night sweats, hypercalcemia, lymphadenopathy, omental thickening, ascitic fluid with a serum ascites albumin gradient of less than 1.1 gm/dL, and exudative pleural effusions. Our suspicion for hematologic malignancy versus disseminated infection was high. During admission, there were many diagnostic challenges in obtaining histologic and bacteriologic confirmation of our leading suspected diagnosis, disseminated tuberculosis. Ultimately, tuberculosis infection was confirmed with histologic evidence of granulomatous inflammation of cervical lymph node and sputum culture positive for *Mycobacterium tuberculosis*. This case highlights the necessity for every patient presenting with new ascites to undergo diagnostic paracentesis. Nephrogenic ascites is a rare syndrome that is possible in volume overloaded states but is a diagnosis of exclusion that should be supported by an exudative serum ascites albumin gradient and no evidence of an alternate etiology.

## 1. Introduction

Nephrogenic ascites is a diagnosis of exclusion in the setting of a serum ascites albumin gradient (SAAG) less than 1.1 gm/dL, meaning portal hypertension is definitively not the cause of the volume overload and that infectious as well as malignant causes of ascites have been ruled out [1, 2]. Anchoring on nephrogenic ascites prematurely can lead to delays in the diagnosis and management of serious illnesses. In the case of our patient, multiple providers had assigned a diagnosis of nephrogenic ascites upon his admission one month earlier. In his case, it had developed in a subacute timeline in the setting of additional signs of systemic illness. The following two serious categories of illness must not be missed when evaluating ascites with a SAAG of less than 1.1 gm/dL: malignancy and infection. We discussed the presentation and diagnosis of a case of disseminated tuberculosis that had initially been attributed to nephrogenic ascites.

Disseminated tuberculosis is a life-threatening illness that presents many diagnostic challenges due to its nonspecific, subtle presentation, which is often underdiagnosed with peritoneal involvement because of this variable presentation, which may be misattributed to volume overload, and the lack of sensitivity in diagnostic studies, including microbiologic analysis of peritoneal fluid. Laboratory markers such as calcium and erythrocyte sedimentation rate, miliary pattern on chest x-ray, and biomarkers such as adenosine deaminase and interferon gamma in fluid study analysis play a role in the workup of suspected tuberculosis. Recently, researchers have studied the utility of using cell-free mycobacterium DNA PCR as an aid in diagnosis; however, the gold standard remains as mycobacterial culture [3, 4].

We described a case that illustrates the diagnostic challenges of disseminated tuberculosis and reinforces the necessity for ascites in patients with end-stage kidney disease to be worked up to identify an underlying cause, given that nephrogenic ascites is a diagnosis of exclusion.

## 2. Case Presentation

A 51-year-old Spanish-speaking male with hypertension and diabetes mellitus complicated by end-stage kidney disease presented to the emergency department for routine hemodialysis with subacute abdominal swelling and discomfort. He endorsed night sweats, 10-lb weight loss, and intermittent diarrhea and constipation over a 6-month duration. He denied shortness of breath, cough, bruising, and vomiting. The patient immigrated from Mexico 24 years earlier and had no exposure to the prison system or housing instability. He was uninsured and relied upon a county hospital compassionate dialysis session twice weekly on Wednesdays and Saturdays through a right subclavian tunneled dialysis catheter.

Notably, the patient's workup had begun in the outpatient setting, five months before his current presentation, when he sought care for the above symptoms, in combination with bilateral pleural effusions, chronic hypoxic respiratory failure (on 1-2 L home oxygen), and cervical lymphadenopathy that were concerning for malignancy. However, the workup had been nondiagnostic. Prior pleural fluid analysis was consistent with an exudative and lymphocytic process with negative acid-fast bacilli (AFB) cultures and negative cytology. Fine needle aspiration of the known cervical lymphadenopathy revealed granulomatous inflammation and both negative cytology and flow cytometry. In the month prior to presentation, he had developed new onset ascites that had been drained and attributed to “nephrogenic ascites” that was attributed to chronic underdialysis given his twice weekly compassionate dialysis and repeated presentations with volume overload. He was admitted to the medicine service for routine dialysis and further workup of ascites.

**Figure 1:**
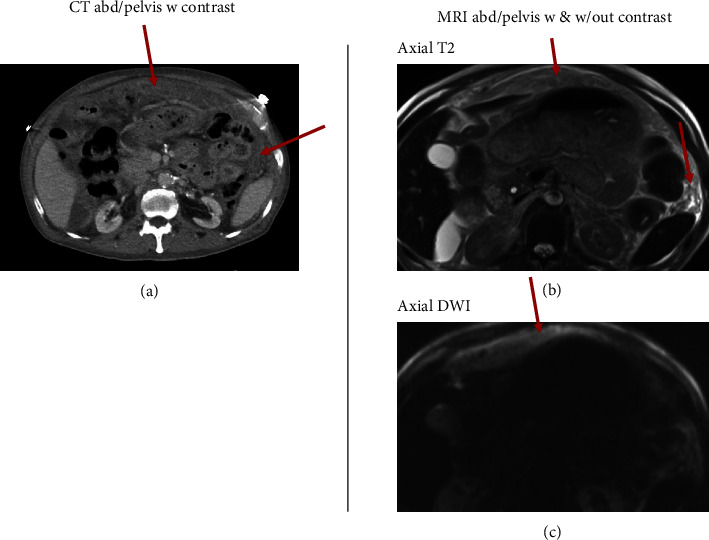
Abdominal imaging upon admission. (a) CT abdomen/pelvis with contrast reveals nodularity in the omentum tracking into the left paracolic gutter (red arrows). MRI abdomen/pelvis with and without contrast shows hyperintense regions on (b) axial T2 weighted sequences and (c) axial diffuse weighted imaging sequences, consistent with areas of inflammation and nodularity (red arrows) with impression including possibility of peritoneal carcinomatosis or infectious process.

On admission, his blood pressure was 160/60 mmHg and his oxygen saturation was 99% on room air. His exam on admission was notable for temporal wasting, anicteric sclera, a moderately distended abdomen with shifting dullness, painless and mobile cervical lymphadenopathy, and decreased basilar breath sounds. Pertinent labs included potassium 5.6 mEq/L, BUN 84 mg/dL, phosphorus 8.3 mg/dL, Cr 10.2 mg/dL, (GFR mL/min/1.73 m^2^), Hgb 10.1, alkaline phosphatase 585 U/L, calcium 13 mg/dL (increased from 8.7 mg/dL six months prior), parathyroid hormone 12 pg/mL, and vitamin D1, 25 hydroxylase 100 ng/mL. Admission EKG showed left ventricular hypertrophy but did not show peaked T waves or any abnormal intervals. Chest x-ray showed prominent pleural effusions. Transthoracic echocardiogram performed 4 months earlier showed normal left ventricular systolic function (ejection fraction 60–65%), grade II diastolic function, with increased left atrial pressure, and normal right ventricular systolic function and no pericardial effusion. Computed tomography of the chest was notable for multistation mediastinal lymphadenopathy, bilateral upper lobe predominant perilymphatic lung nodules, and bilateral pleural effusions with pleural thickening, suggestive of pulmonary sarcoidosis with a differential of atypical mycobacterial infections. Radiology noted that nodularity and hyperenhancing lymphadenopathy that was unusual for disseminated mycobacterial tuberculosis or histoplasmosis given lack of miliary pattern. Computed tomography of the abdomen and pelvis demonstrated anasarca, large volume abdominopelvic ascites with peritoneal thickening, and nodularity throughout the omentum (Figure 1(a)). MRI abdomen/pelvis with contrast showed enhancing soft tissue thickening throughout the greater omentum (Figures 1(b) and 1(c)) and in their impression suggested the possibility of malignancy with peritoneal carcinomatosis with recommendation for tissue sampling.

Based upon this patient's ongoing outpatient workup and new imaging findings, there was skepticism that the ascites was solely attributable to inadequate dialysis. For both diagnostic and therapeutic purposes, paracentesis was performed. The ascitic fluid analysis revealed a serum ascites albumin gradient of less than 1.1 gm/dL, which was not consistent with an etiology secondary to elevated hydrostatic pressure. Additionally, fluid studies indicated an elevated adenosine deaminase level of 32.6 U/L but a negative AFB stain. Serum interferon gamma release assay was positive. Core biopsy of cervical lymph node revealed granulomatous inflammation with central necrosis and also negative AFB stain. Bronchoscopy was unrevealing. AFB stain and MTB PCR were positive on sputum sample, eventually growing pan-sensitive*Mycobacterium tuberculosis*. Patient was initiated on RIPE therapy (i.e., rifamycin, isoniazid, pyrazinamide, and ethambutol) empirically after bronchoscopy was complete. Given the patient's constellation of symptoms, hypercalcemia with evidence of granulomatous disease, and imaging findings of omental caking and pulmonary disease, a final diagnosis of disseminated tuberculosis was made with confirmed sputum sample.

## 3. Discussion

A new presentation of ascites should prompt the clinician to perform paracentesis if there is sufficient fluid to pursue a paracentesis. If the patient has spontaneous bacterial peritonitis, studies suggest that each hour delay in paracentesis correlates to a 3% per hour mortality risk increase [5]. Nephrogenic ascites occurs in patients on hemodialysis, with proposed mechanisms including elevated hepatic vein hydrostatic pressure (without portal hypertension) and impaired lymphatic peritoneal resorption [1].

From the emergency department perspective, this patient required admission for dialysis due to volume overload; “nephrogenic ascites” had been documented in the patient's chart at a prior emergency department visit for dialysis. Verbal sign-out documentation indicated that routine dialysis was indicated and that the patient was chronically under dialyzed and volume overloaded secondary to constraints with the schedule of compassionate dialysis.

However, the following several features of this patient's case pointed to a systemic process: hypercalcemia, diffuse lymphadenopathy, perilymphatic nodules on CT chest, and nodularity of omentum tissue. In his case, paracentesis was diagnostic and therapeutic. His SAAG was less than 1.1 gm/dL, supporting an exudative process [6]. Because tuberculosis is endemic in his home country of Mexico, tuberculosis was high in the differential diagnosis. Direct AFB smear on the ascites fluid has a 2% sensitivity for mycobacterium [7], while sensitivity of mycobacterial culture ranges from 62% to 83% [7, 8]. Adenosine deaminase (ADA) can be a useful lab to include given that its sensitivity approaches 58% with specificity 95% in patients without cirrhosis [9]. Koff and Azar [10] observed that several studies have found an ADA value of >30 IU/L in ascitic fluid to be both highly sensitive and specific for peritoneal tuberculosis. ADA is a purine-degrading enzyme expressed by T lymphocytes in response to antigenic stimulation and appears to be upregulated in CD4 T cells responding to mycobacterial antigen stimulation relative to malignant lymphocytes [11]. In this patient, the elevated ADA level suggested a CD4-predominant immune response, as is the case for granulomatous disease. This patient was found to have *M*. *tuberculosis* by PCR of sputum sample and was started on RIPE prior to discharge.

In cases of suspected peritoneal tuberculosis, ascites fluid is exudative and lymphocytic, and stains for AFB have low sensitivity; inclusion of ADA in ascites fluid can support a diagnosis of granulomatous inflammation. When positive, ascitic fluid mycobacterial culture is diagnostic of peritoneal tuberculosis. If culture is negative, but suspicion remains high, the gold standard and next steps in diagnosis should include laparoscopy and peritoneal biopsy for histologic diagnosis [12].

Transitions from the ambulatory setting and emergency department to inpatient care present an opportunity for medical errors to occur or for potentially incorrect diagnoses to persist if alternate etiologies are not considered. The physician receiving a handoff at any transition should strive to avoid cognitive bias such as anchoring bias based upon the initial sign-out, given that anchoring bias is amongst the cognitive errors most associated with adverse patient outcomes [13].

In summary, every patient presenting with new ascites should undergo diagnostic paracentesis. Nephrogenic ascites is a rare syndrome that is possible in volume overloaded states but is a diagnosis of exclusion that should be supported by an exudative SAAG and no evidence of an alternate etiology.

## Data Availability

Additional case information used to support the findings of this study have not been made available in order to protect patient privacy.
